# COE2 Is Required for the Root Foraging Response to Nitrogen Limitation

**DOI:** 10.3390/ijms23020861

**Published:** 2022-01-13

**Authors:** Rui Wu, Zhixin Liu, Jiajing Wang, Chenxi Guo, Yaping Zhou, George Bawa, Jean-David Rochaix, Xuwu Sun

**Affiliations:** 1 State Key Laboratory of Crop Stress Adaptation and Improvement, State Key Laboratory of Cotton Biology, Key Laboratory of Plant Stress Biology, School of Life Sciences, Henan University, 85 Minglun Street, Kaifeng 475001, China; wuruiwr347538@sina.com (R.W.); zhixinliuhenu@sina.com (Z.L.); wdj_3079@sina.com (J.W.); chenxi1445@sina.com (C.G.); zhouyapinghenu@sina.com (Y.Z.); ge.9410@yahoo.com (G.B.); 2 Departments of Molecular Biology and Plant Biology, University of Geneva, 1211 Geneva, Switzerland; jean-david.rochaix@unige.ch

**Keywords:** nitrogen, COE2, RNA-seq, root, nitrogen limitation

## Abstract

There are numerous exchanges of signals and materials between leaves and roots, including nitrogen, which is one of the essential nutrients for plant growth and development. In this study we identified and characterized the Chlorophyll A/B-Binding Protein (*CAB*) (named *coe2* for *CAB* overexpression 2) mutant, which is defective in the development of chloroplasts and roots under normal growth conditions. The phenotype of *coe2* is caused by a mutation in the Nitric Oxide Associated (*NOA1*) gene that is implicated in a wide range of chloroplast functions including the regulation of metabolism and signaling of nitric oxide (NO). A transcriptome analysis reveals that expression of genes involved in metabolism and lateral root development are strongly altered in *coe2* seedlings compared with WT. *COE2* is expressed in hypocotyls, roots, root hairs, and root caps. Both the accumulation of NO and the growth of lateral roots are enhanced in WT but not in *coe2* under nitrogen limitation. These new findings suggest that COE2-dependent signaling not only coordinates gene expression but also promotes chloroplast development and function by modulating root development and absorption of nitrogen compounds.

## 1. Introduction

Leaves and roots mutually regulate each other’s development [1]. Leaves provide energy for root development through photosynthesis and water absorption through respiration [2]. There is an extensive exchange of material and information between leaves and roots [3]. Because leaves also contain many mineral nutrients, they strongly depend on the absorption and supply of these metabolic raw materials by roots [4]. In leaves, chloroplasts are the center organelles for photosynthesis and metabolic reactions [5,6,7]. Chloroplasts rely on plastid retrograde signaling to regulate the expression of nuclear genes involved in seedling development [6,7]. As an example, our previous study revealed that the *coe1* mutant is defective in the development of both chloroplasts and whole seedlings [8].

Nitrogen is one of the essential nutrients for plant growth and development [9]. It is an important component of chlorophyll, amino acids, nucleic acids, and secondary metabolites [10,11]. At the same time, nitrogen also acts as a signal for regulating plant growth and development [12]. Nitrogen deficiency strongly inhibits plant growth and development resulting in the inhibition of leaf and root development. It induces chlorophyll degradation and affects other metabolic processes [13,14]. The demand for nitrogen by leaves is satisfied through the absorption of nitrogen compounds by roots. At the same time, photosynthesis and respiration in leaves can provide energy and power for the absorption of these compounds by roots [2]. In addition, when plants are suffering from nutritional deficiencies such as nitrogen and phosphate, the foraging response of plant roots is activated to enhance the absorption of mineral nutrients from soils [15,16]. Some studies have shown that stress responses or signals from leaves induce root foraging responses [17]. However, the signal pathways coordinating the nitrogen requirement in leaves with the root foraging response are still unclear.

In this study, we characterized the *CAB overexpression* (*coe*) *2* mutant, which is defective in developing chloroplasts and roots. A Bulked Segregant Analysis (BSA) showed that one STOP mutation in the Nitric Oxide Associated (*NOA1*) gene is responsible for the phenotype of *coe2*. RNA-seq analysis revealed that the transcriptome profile is significantly changed in the *coe2* mutant. Furthermore, root development in response to nitrogen limitation in *coe2* was also affected. These new findings suggest that COE2-dependent signaling not only coordinates gene expression but also promotes chloroplast development and function by modulating root development and absorption of nitrogen compounds. 

## 2. Results

### 2.1. Isolation and Identification of coe2 Mutant

**Figure 1:**
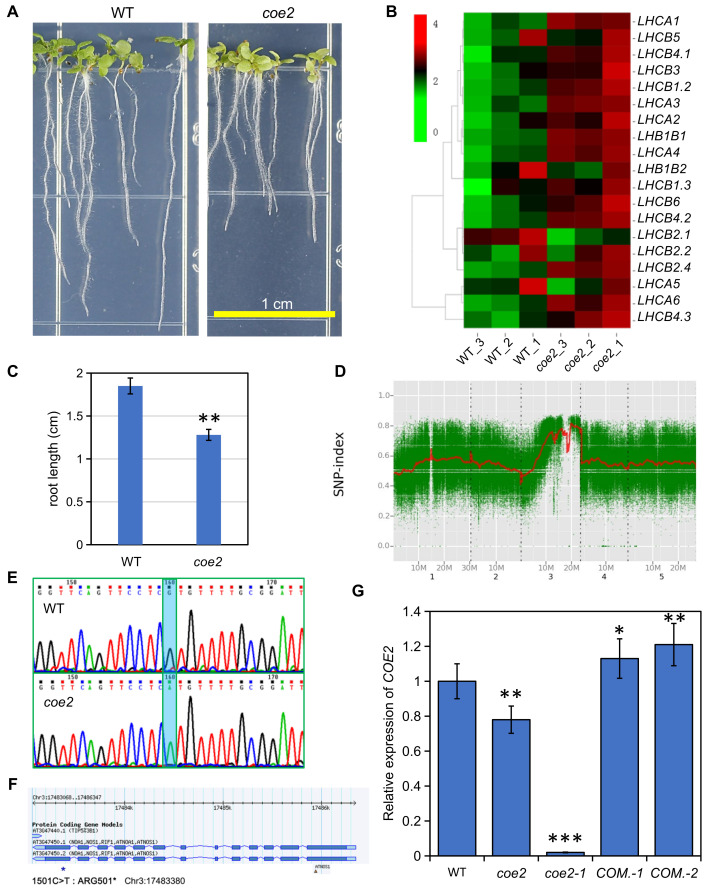
Identification of *coe2* mutant. (**A**) Phenotype of 5-day-old seedlings of *coe2* and WT on 1/2 MS plates. (**B**) Heatmap of the expression of genes of light-harvesting proteins in cotyledons of seedlings of *coe2* and WT under normal growth conditions. (**C**) Statistical analysis of the root length in 5-day-old seedlings of WT and *coe2*. Three biological replications were used. The student’s *t*-test of *coe2* versus WT, ** *p* < 0.001. (**D**) The SNP-index of *coe2*. SNPs between *coe2* and the reference genome were analyzed by Bulked Segregant Analysis. (**E**) The candidate mutation of *coe2* was identified by DNA sequencing. (**F**) The mutation of *coe2* (blue star) and *coe2-1* (triangle, SALK_047882) are marked on the gene map. (**G**) Relative expression of COE2 in *coe2*, *coe2-1*, and WT was determined by qPCR. Three biological replicates were used. The student’s *t*-test of *coe2* or *COM.* Versus WT, * *p* < 0.05%, ** *p* < 0.01%, *** *p* < 0.001.

**Figure 2:**
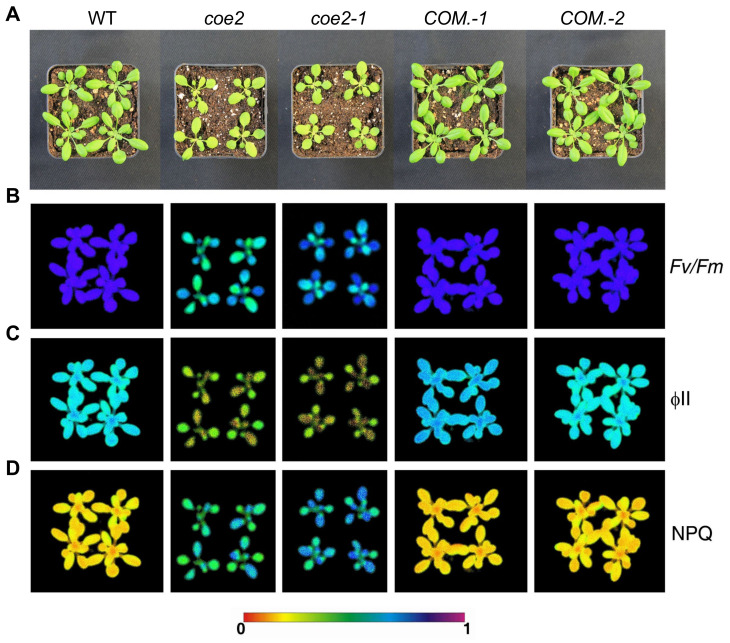
*coe2* is defective in photosynthesis. (**A**) Growth of *coe2*, *coe2-1*, complemented plants of *coe2-1* (*COM.-1*), *COM.-2*, and WT in soil for four weeks. (**B**) Fv/Fm, (**C**) ΦII, and (**D**) NPQ for the seedlings shown in (**A**) were measured with an imaging PAM as described in Materials and Methods.

To study the physiological function of plastid retrograde signaling, we generated a mutant library of *Arabidopsis thaliana* using EMS mutagenesis [8]. A series of *coe* mutants overexpressing *CAB* genes were isolated under normal growth conditions [8]. The *coe2* mutant was selected for a systematic characterization in this study. Cotyledons of *coe2* seedlings were pale-yellow (Figure 1A and Figure 2A). An analysis of chlorophyll fluorescence of WT and *coe2* plants indicated that Fv/Fm and PSII photochemical efficiency (ΦPSII) were reduced in the mutant (Figure 2B,C). The expression of differentially expressed genes (DEGs) of cotyledons of seedlings of *coe2* and WT was observed using heat map analysis (Figure 1B).

In contrast, non-photochemical quenching (NPQ) in *coe2* was significantly increased (Figure 2D). We analyzed the chloroplast ultrastructure in *coe2* through transmission electron microscopy (TEM) to analyze the effects of the mutation on chloroplast development and function. The chloroplast development of *coe2* was slightly affected compared with WT; in particular, grana thylakoids were sparse and thinner (Figure S1A–C). We then extracted total proteins from the seedlings of WT and *coe2* and examined chloroplast protein accumulation using immunoblotting. The levels of most subunits of the photosystem complexes were reduced in *coe2* compared with WT (Figure S1D). Further, we extracted thylakoid membranes from WT and *coe2* and analyzed the photosystem complexes by BN-PAGE (Figure S2). Accumulation of dimer complexes and supercomplexes of PSII and PSI were slightly reduced in *coe2* compared with WT (Figure S2). Besides chloroplast development, root development was also impaired in *coe2* (Figure 1A,C) suggesting that *coe2* may play a role in this process. The data also indicate that *coe2* is an EMS mutant, not affecting its expression but its function. 

### 2.2. Mutation in the NOA1 Gene Causes the Phenotype of coe2 

To identify the mutation site of *coe2*, we first backcrossed the *coe2* mutant with the WT (Col ecotype) for three generations. A statistical analysis indicated that the proportion of seedlings with a similar phenotype as *coe2* in the backcrossed progeny was 23~26%. Subsequently, we crossed *coe2* and Ler (wild type) to produce a mapping population. In the progeny population of *coe2* × Ler, the ratio of seedlings showing a similar phenotype as *coe2* was around 1:4. Three hundred plants with the *coe2* phenotype were selected from this progeny population and were pooled for genomic DNA extraction. The extracted DNA was first used to construct a next-generation sequence (NGS) library for next-generation sequencing. We also carried out NGS for the Col and Ler genomes, which were used as reference genomes for the Bulked Segregant Analysis (BSA). Subsequently, the sequencing results were processed according to the BSA method. Ultimately, we identified a mutation from C to T in nucleotide 1501 of the gene *AtNOA1* [18,19] resulting in a stop codon that leads to premature termination of translation (Figure 1D,E). *AtNOA1* is involved in regulating nitric oxide (NO) signaling [18,19]. To verify that this mutation is responsible for the phenotype of *coe2*, we screened T-DNA insertion lines and identified one with an insertion in the *NOA1* gene (SALK_047882: herein named *coe2-1*) (Figure 1F). Under normal growth conditions, *coe2-1* and *coe2* exhibited similar growth phenotypes and chlorophyll fluorescence patterns (Figure 2). We introduced the wild-type *coe2* cDNA into *coe2* and generated complemented transgenic plants. The wild-type phenotype and chlorophyll fluorescence were nearly fully restored in the complementation (*COM*.) lines (Figure 2). Further, we analyzed the levels of *COE2* mRNA in *coe2* and *coe2-1* by qRT-PCR. Almost no *COE2* expression was detected in *coe2-1* compared with WT (Figure 1G), and the expression level of *COE2* in *coe2* was decreased by 75% compared with WT (Figure 1G). Taken together, these findings indicate that the mutation in *coe2* is responsible for the defects of the *coe2* mutants. 

**Figure 3:**
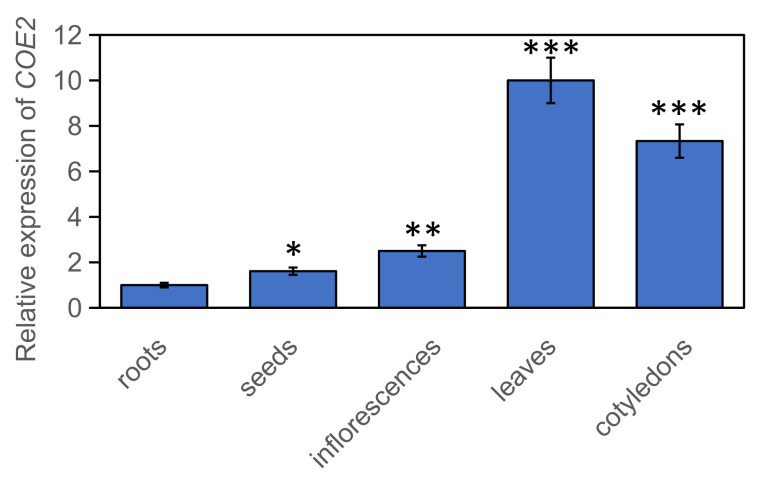
Expression patterns of *COE2* in different tissues of seedlings. The samples of cotyledons, roots, mature leaves, inflorescence, and seeds were harvested and used to examine the expression of *COE2* by qPCR. Three biological replicates were used. The student’s *t*-test of other tissues versus roots, * *p* < 0.05%, ** *p* < 0.01%, *** *p* < 0.001%.

We then performed qPCR to examine the expression of *COE2* in different tissues. Consistent with its potential role in the regulation of root development, the expression of *COE2* can be detected in cotyledons, roots, mature leaves, inflorescence, and seeds (Figure 3). The expression of *COE2* in roots is consistent with the previous reports [20]. As reported by others, the NO levels in roots of *coe2* were similar to WT [21]. Although no significant difference of NO accumulation was observed between *coe2* and WT, NO-dependent signaling was defective in *coe2* [22]. Therefore, a potential role of COE2-mediated NO signaling in the regulation of root development is possible. 

### 2.3. COE2 Is Involved in Regulating the Expression of Genes Involved in Root Development

**Figure 4:**
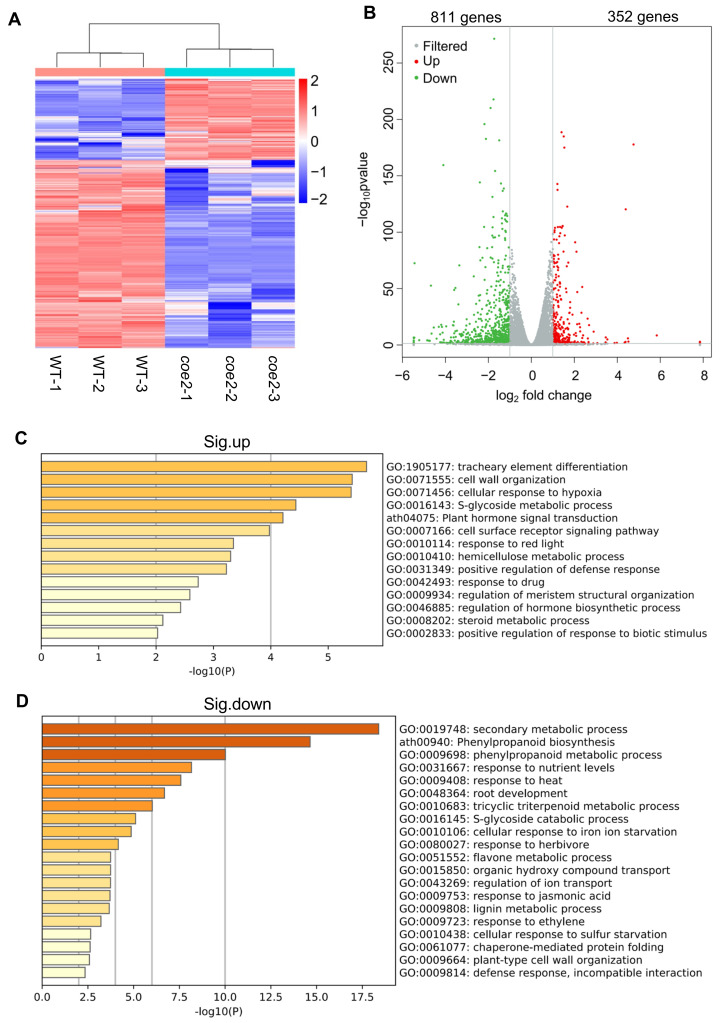
Analysis of the differentially expressed genes (DEGs) in cotyledons of *coe2* and WT. (**A**) Heatmap displays the expression pattern of the DEGs in cotyledons of *coe2* and WT. (**B**) Volcano plot shows the distribution of the DEGs in cotyledons of *coe2* and WT. Gene Ontology (GO) analysis of the up-regulated DEGs (**C**) and significantly down-regulated DEGs (**D**) in cotyledons of *coe2*, compared with WT.

To further dissect the mechanism by which COE2 regulates plant growth and development, we performed an RNA-sequence (RNA-Seq) analysis separately on cotyledons and roots of 7-day-old seedlings of *coe2* and WT grown on plates containing ½ Murashige and Skoog (MS) medium. As shown in Figure 4A, 1163 differentially expressed genes (DEGs) were identified in cotyledons of *coe2* seedlings, 811 genes were significantly down-regulated, whereas 352 genes were significantly up-regulated (Figure 4A,B and Table S1). A Gene Ontology (GO) analysis indicated that the up-regulated DEGs were mainly involved in tracheary element differentiation, cell wall organization, and response to red light (Figure 4C). In contrast, genes involved in secondary metabolism, phenylpropanoid biosynthesis, response to ethylene (ETH) and jasmonic acid (JA), and root development were significantly enriched amongst the down-regulated DEGs (Figure 4D). It is well-known that ETH and JA play important roles in regulating the development of roots [23,24]. Consistent with the down-regulation of genes related to JA, the growth of *coe2* and *coe2-1* roots showed increased sensitivity to JA (Figure S3). Thus, the retarded root development in *coe2* may be due to decreased expression of genes for ETH or JA. The expression of DEGs related to root development was impaired in cotyledons of *coe2,* suggesting that COE2 may control root development by regulating the expression of these genes in cotyledons.

**Figure 5:**
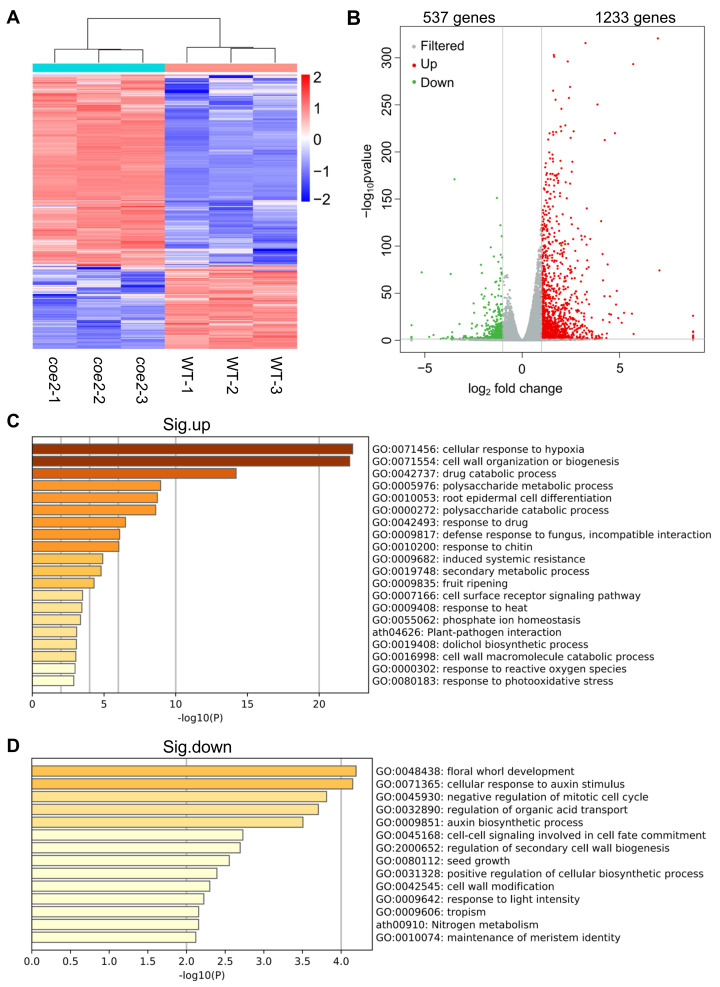
Analysis of the differentially expressed genes (DEGs) in roots of *coe2* and WT. (**A**) Heatmap displays the expression pattern of the DEGs in roots of *coe2* and WT. (**B**) Volcano plot shows the distribution of the DEGs in cotyledons of *coe2* and WT. Gene Ontology (GO) analysis of the up-regulated DEGs (**C**) and significantly down-regulated DEGs (**D**) in roots of *coe2* compared with WT.

In the roots of *coe2*, 1870 DEGs were identified, and 1233 genes were significantly up-regulated, while 537 genes were significantly down-regulated (Figure 5A,B and Table S2). A GO analysis indicated that the expression of genes for cellular response to hypoxia, cell wall organization and biogenesis, root epidermal cell differentiation, polysaccharide metabolism, secondary metabolism, and cell surface receptor signaling pathways were significantly up-regulated (Figure 5C). These results suggest that the genes for metabolic processes were activated in the roots of *coe2*. In contrast, the down-regulated DEGs were mainly related to floral whorl development, cellular response to auxin stimulus, cell-cell signaling involved in cell fate commitment, and the regulation of secondary cell wall biogenesis (Figure 5D). These results suggest that COE2 may inhibit the expression of genes for root cell differentiation while enhancing the expression of genes for cell fate commitment and cell wall biogenesis. In addition, the expression of genes involved in the negative regulation of the mitotic cell cycle and maintenance of meristem identity was also down-regulated (Figure 5D). Both cell cycle and meristem identity are required for root development [25,26,27,28,29,30], suggesting COE2 may play important roles in regulating root development. Interestingly, we found that the genes involved in the cellular response to hypoxia, cell wall organization and biogenesis, and the cell surface receptor signaling pathway were significantly up-regulated in both cotyledons and roots of *coe2* (Figure 4C and Figure 5C), suggesting that the regulation of these genes by COE2 is not limited to the tissue type. To assess the data of RNA-seq, we detected the expression of *PIF4* in cotyledons and roots of seedlings by qPCR. As shown in Figure S4, the expression of *PIF4* detected by qPCR is consistent with that of RNA-seq.

To assess the potential roles of COE2-dependent shoot signaling in the development of roots, we performed grafting experiments using the shoots and roots from WT, *coe2*, and *coe2-1* mutants. As shown in Figure S5A, the development of roots in grafted seedling of WT-shoot/WT-root, *coe2*-shoot/*coe2*-root, *coe2-1*-shoot/*coe2-1*-root was similar to WT and *coe2*. As expected, the development of roots in grafted seedling of *coe2*-shoot/WT-root and *coe2-1*-shoot/WT-root was impaired while this process was enhanced in grafted seedling of WT-shoot/*coe2*-root and WT-shoot/*coe2-1*-root (Figure S5B). These results suggest that COE2-dependent shoot signaling is required for the regulation of root development.

### 2.4. Nitrogen Limitation Affects the Root Development

RNA-seq analysis further revealed that the expression of genes for nitrogen metabolism in roots of *coe2* was also impaired (Figure 5D). Nitrogen metabolism is required not only for leaf but also for root development [31,32]. Therefore, the effects of *coe2* on the expression of genes for nitrogen metabolism indicated that COE2 might be required for the regulation of root development under nitrogen limitation. To dissect the mechanism by which COE2 regulates the development of roots, we first investigated the expression patterns of *COE2* under normal conditions. As shown in Figure 3, the expression of *COE2* was detected in cotyledons, hypocotyl, root tips, and lateral roots of seedlings. The expression of *COE2* in roots further supports the potential role of COE2 in regulating the expression of genes for root development. 

### 2.5. COE2 Is Involved in Regulating Root Development in Response to Nitrogen Limitation

**Figure 6:**
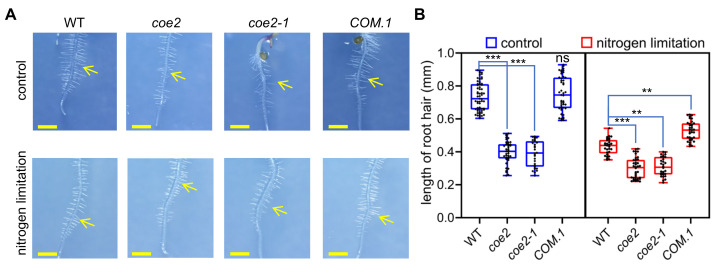
COE2 is involved in the regulation of root hair development in response to nitrogen availability. (**A**) Root hair growth status in *coe2*, *coe2-1*, *COM.-1*, and WT grown on 1/2MS plates (control conditions) and 1/2MS plates containing 0.1 mM KNO3 (nitrogen limitation). Root hairs are indicated with yellow arrows. (**B**) Whisker box analysis of the length of root hairs in *coe2*, *coe2-1*, *COM.-1*, and WT grown under control and nitrogen limitation conditions. The data were analyzed by one-way ANOVA following Brown–Forsythe test. Ns: *p* > 0.05, ** *p* < 0.01, *** *p* < 0.001.

The development of root hairs in *coe2* and *coe2-1* was significantly altered compared with WT (Figure 6A). Under nitrogen limitation, the development of root hairs was significantly inhibited in WT, but not in *coe2* and *coe2-1* (Figure 6A,B), suggesting that nitrogen is required for the development of root hairs and COE2 is involved in regulating the root hair development in response to nitrogen limitation. 

**Figure 7:**
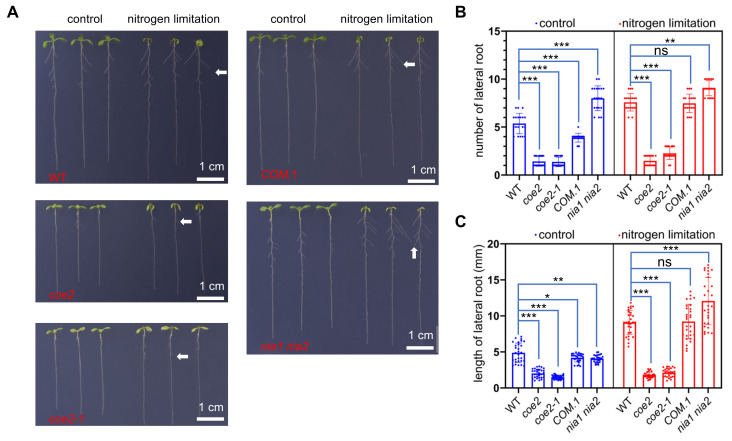
COE2 is involved in regulating the development of lateral roots under nitrogen limitation conditions. (**A**) Growth of seedlings of WT, *coe2*, *coe2-1*, *COM.1*, and *nia1 nia2* under control and nitrogen limitation conditions. The lateral roots are indicated with white arrows. (**B**) Whisker box analysis of the number of root hairs in WT, *coe2*, *coe2-1*, *COM.-1*, and *nia1 nia2* grown under control and nitrogen limitation conditions. (**C**) Whisker box analysis of the length of root hairs in WT, *coe2*, *coe2-1*, *COM.-1*, and *nia1 nia2* grown under control and nitrogen limitation conditions. The data were analyzed by one-way ANOVA following Brown–Forsythe test. Ns: *p* > 0.05, * *p* < 0.05, ** *p* < 0.01, *** *p* < 0.001.

The growth of cotyledons and leaves of WT seedlings was seriously impaired under nitrogen limitation, but the growth of lateral roots was enhanced (Figure 7A–C). Compared with WT, the growth of cotyledons and leaves of *coe2* mutant seedlings was barely inhibited, and the growth of lateral roots was not significantly increased under nitrogen limitation (Figure 7A). These results indicate that COE2 regulates the signaling response to nitrogen limitation for the growth of cotyledons, leaves, and lateral roots. NIA1 and NIA2 are involved in the regulation of nitrogen uptake and NO production. The *nia1 nia2* double mutant was more sensitive to nitrogen limitation (Figure 7A,B). Under this condition, compared with WT, the growth of cotyledons and leaves of *nia1 nia2* double mutant was more inhibited, but the growth of lateral roots was significantly increased. These results suggest that nitrogen limitation signaling is involved in regulating the growth of cotyledons, leaves, and lateral roots, suggesting that COE2 may be implicated in regulating the perception of the signal elicited by nitrogen limitation.

To further evaluate the effect of *coe2* on plant growth under nitrogen limitation, seedlings were grown under normal conditions and nitrogen limitation on MS medium for 4 weeks (Figure S5). The results showed that the growth level of leaves and roots of the *coe2* mutant were significantly lower under normal growth conditions than WT. Under nitrogen limitation, the growth of leaves in WT was strongly inhibited, while this inhibition was weaker in *coe2*. Compared with the strong inhibition of leaf growth, nitrogen limitation had less effect on root growth (Figure S6A,B). Under nitrogen limitation, root development of *coe2* mutant was still slower compared with WT. Compared with WT, leaf growth of the *nia1 nia2* double mutant was more inhibited, but root growth was enhanced (Figure S6A,B). These results indicate that the nitrogen limitation signal is strongly activated in the *nia1 nia2* double mutant. They also indicate that nitrogen limitation can promote lateral root development but inhibit leaf development. At the same time, these phenomena also suggest that COE2 may be involved in regulating the sensitivity to nitrogen limitation.

### 2.6. COE2-Dependent Signaling Regulates Root Development by Controlling the Expression of Downstream Transcription Factors

**Figure 8:**
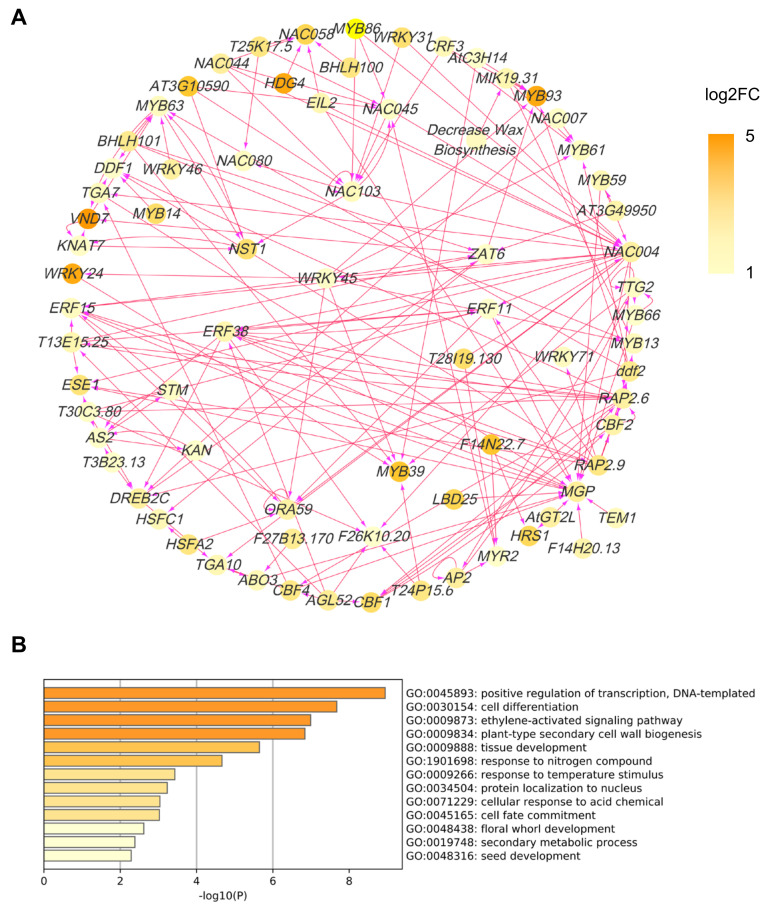
Analysis of the transcription factor (TF) network of the DEGs in the cotyledons of *coe2*. (**A**) The TFs of DEGs of cotyledons of *coe2* were selected to build the TF regulatory network. The color key indicates low to high gene expression levels (log2 FC). (**B**) GO analysis of the TFs shown in (**A**).

**Figure 9:**
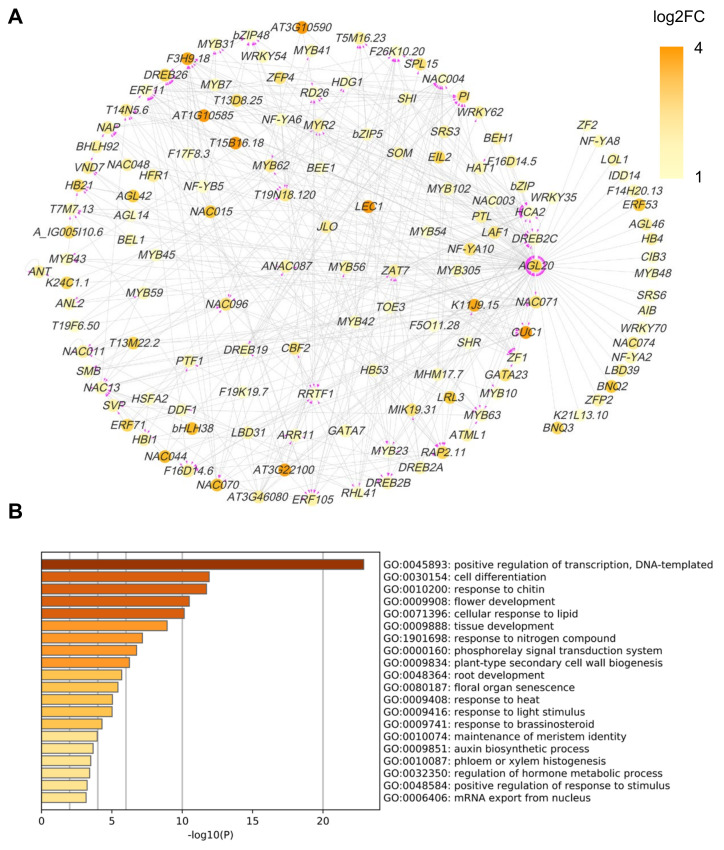
Analysis of the transcription factor (TF) network of the DEGs in the roots of *coe2*. (**A**) The TFs of DEGs of cotyledons of *coe2* were selected to build the TFs regulatory network. The color key indicates low to high gene expression levels (log2 FC). (**B**) GO analysis of the TFs shown in (**A**).

To address the downstream network of COE2-dependent signaling, we constructed a transcription factor (TF) regulatory network for DEGs in cotyledons and roots. For cotyledons, the network was mainly composed of WRKY, NAC, and MYB family TFs (Figure 8A). GO analysis indicated that these TFs were mainly involved in positive regulation of transcription and cell differentiation (Figure 8B). As expected, some TFs were involved in regulating the response to nitrogen compounds (e.g., ZAT6, MYB59, and HRS1) (Figure 8B) [33,34,35,36,37]. Amongst those, HRS1 and ZAT6 are essential for the metabolism of both nitrogen and phosphate and root development [33,36,37,38]. ZAT6 plays an important role in regulating root development and phosphate (Pi) acquisition and homeostasis and may act as a repressor of primary root growth and regulate Pi homeostasis through the control of root architecture [37]. HRS1 is involved in nitrate and phosphate signaling in roots and plays an important role in integrating nitrate and phosphate starvation responses and adaptation of root architecture depending on nutrient availability [33,36]. HRS1 acts downstream of the nitrate sensor and transporter NPF6.3/NRT1.1 [33]. It is required for the modulation of primary root and root hair growth under phosphate deprivation [36]. In the presence of nitrate, HRS1 can repress primary root development in response to phosphate depletion [36]. For roots, a GO analysis indicated that some TFs are directly involved in regulating root development (e.g., CUC1, AGL14, SOMBRER (SMB), and LRL3) [39,40,41,42] (Figure 9A,B). On the other hand, some TFs can regulate root development through their effects on cell differentiation, phloem or xylem histogenesis, and auxin biogenesis (Figure 9B). In addition, some TFs are also involved in regulating the response to nitrogen compounds (e.g., RRTF1, PTL, and AZF1(ZF1)) [43,44,45,46] (Figure 9A,B), suggesting that COE2-dependent signaling can rely on these TFs to regulate nitrogen metabolism for facilitating root development.

## 3. Discussion

### 3.1. COE2 Is Involved in Regulating Plastid Retrograde Signaling 

Chloroplasts are not only the center of metabolism and key biochemical reactions but also the central hub for sensing environmental information. Therefore, the development and function of chloroplasts are very sensitive to internal (e.g., phytohormone and metabolites) and external influences (e.g., temperature and light intensity) [47,48,49,50]. The organelle communication network plays an important role in coordinating the expression of nuclear genes for maintaining the development and function of chloroplasts under both normal and adverse growth conditions [8]. Surprisingly *coe2* is defective not only in plastid retrograde signaling but also in the regulation of other nuclear genes that are involved in the root development of seedlings. 

A BSA and genetic complementation analysis revealed that the phenotype of *coe2* is caused by a mutation in Nitric Oxide Associated (*NOA1*) (Figure 1). *NOA1* is involved in regulating the metabolism and signaling pathway of NO [21,51,52,53]. NO is a small, water and lipid-soluble gas that has emerged in recent years as a major signaling molecule of ancient origin and ubiquitous importance [54,55,56,57]. *NOA1* localization in chloroplasts of cotyledons [20] suggests that COE2-mediated NO signaling may act as one kind of plastid retrograde signaling to regulate the expression of nuclear genes. 

### 3.2. COE2-Dependent Signaling Is Involved in Regulating the Foraging Response to Nitrogen Limitation 

One interesting result of our study is that *coe2* is defective in root development (Figure 1A). Nitrogen metabolism is required for root development [31,58,59,60]. In the nitrogen metabolism pathway, two key enzymes NIA1 and NIA2, are also required to produce NO [53,61,62] indicating that nitrogen metabolism is required for the generation of NO. A recent report suggests that auxin produced in leaves is involved in regulating root development in response to pH status and nitrogen availability [62]. The growth of the aerial part of plants is closely linked to the status of nitrogen assimilation. Nitrogen metabolites are mainly absorbed by roots and transported to the leaves [3]. The demand for nitrogen in leaves may stimulate its absorption in roots. However, it is not clear how the signals generated by nitrogen limitation in leaves are transmitted to roots. A GO analysis indicated that some DEGs in roots of *coe2* are linked to nitrogen metabolism (Figure 8B) suggesting that COE2 may modulate root development by regulating nitrogen metabolism directly or indirectly. Expression of COE2 within root maturation zones and root hairs suggests that COE2 may influence the development of root hairs by regulating the absorption of nitrogen compounds (Figure 3). The development of root hairs was impaired in the *coe2* and *coe2-1* mutants but rescued in *COM*. Transgenic plants under nitrogen limitation. *Coe2* seedlings grown on MS medium plates and in soil grew significantly slower than WT. These results suggest that COE2-dependent NO signaling is required for the development of roots in response to nitrogen limitation.

### 3.3. COE2 Regulates the Development of Roots in Response to Nitrogen Limitation through Down-Stream TFs

**Figure 10:**
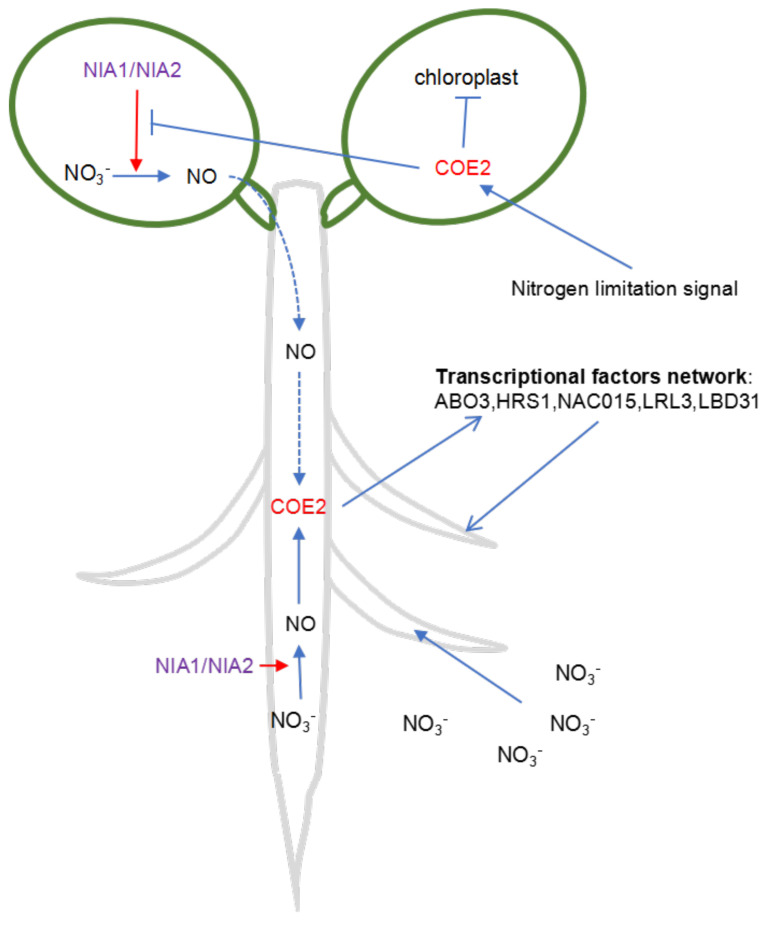
Model showing how COE2 regulates root development in response to nitrogen limitation. Under nitrogen limitation, once COE2 perceives the nitrogen limitation signal in leaves, production of NO regulated by NIA1/NIA2 is triggered. At the same time, COE2 is involved in the inhibition of chloroplast and leaf development in response to the nitrogen limitation signals. Under the conditions of nitrogen limitation, NO is produced in leaves and transported to roots to enhance the expression of TFs and development of lateral roots by COE2; alternatively, NO is directly produced in roots and then relies on COE2 to regulate the expression of TFs and the development of lateral roots.

Transcription factors and their network are responsible for regulating gene expression in response to internal or external signals. We constructed a TF regulatory network based on the DEGs in cotyledons and roots of *coe2* (Figure 8 and Figure 9). In this TF regulatory network, the TFs also regulate each other. Consistent with potential functions of COE2, TFs for the response to nitrogen compounds, cell differentiation, and cell wall biogenesis were active in the cotyledon TF network, while TFs for root development, phloem and xylem histogenesis, and the auxin biosynthetic process were active in the root TF network. These results suggest that COE2 may rely on the downstream TF network to regulate the absorption and assimilation of nitrogen and the development of roots (Figure 10). Although NO’s potential roles in the regulation of nitrogen metabolism and root development have been proposed previously [31], characterization of the TF regulatory network and the identification of key TFs have not been thoroughly explored. Based on the best-characterized function of some central TFs in our TF regulatory network, we can tentatively outline a COE2-mediated signaling pathway and the downstream TF regulatory network. These results provide a blueprint for further systematic characterization of the process by which COE2 regulates the absorption and assimilation of nitrogen compounds and the development of roots. Our studies suggest that COE2 is involved in the perception of limited nitrogen availability and the regulation of seedling development under nitrogen limitation. 

## 4. Materials and Methods

### 4.1. Screening and Characterization of Mutants

The *coe2* mutant was identified by screening for the *coe* phenotype from the EMS population. The T-DNA insertional mutants, *coe2-1* (SALK_047882) and *nia1 nia2* (*nia1-1*; *nia2-5*) were obtained from the Arabidopsis Biological Resource Center (ABRC). Mutant lines homozygous for the T-DNA insertion were isolated by PCR analysis using gene-specific and T-DNA-specific primers (Table S3). In addition, we generated the complemented lines of *coe2* (in the background of *coe2*). 

### 4.2. Constructs for Plant Transformation

To generate the complementation constructs, the full-length cDNA of *COE2* was PCR-amplified using the primer pairs as described in Table S1. Then the PCR products were purified and first cloned into pDNOR201 by BP Clonase reactions (Gateway^®^ BP Clonase^®^ II, Invitrogen, ThermoFisher Scientific, Waltham, MA, USA) according to the manufacturer’s instructions to generate the pDNOR-COE2. The resulting plasmids were recombined into pB7WGF2 using LR Clonase reactions (Gateway^®^ LR Clonase^®^ II, Invitrogen, ThermoFisher Scientific, Waltham, MA, USA) to generate the final constructs. 

### 4.3. Plant Transformation

The complementation construct was transformed into *Agrobacterium tumefaciens* strain GV3105 via electroporation. Then the *Agrobacterium tumefaciens* that contained the complementation construct was introduced into *coe2*. The resulting T1 transgenic plants were selected by BASTA as described previously [8]. Homozygous transgenic plants were used in all experiments. 

### 4.4. Chlorophyll (Chl) Fluorescence Analysis

In vivo Chl *a* fluorescence of whole seedlings was recorded using an imaging Chl fluorometer (ImagingPAM; Walz, Germany). To measure Fv/Fm, dark-adapted plants were exposed to a pulsed, blue measuring beam (1 Hz, intensity 4; F0) and a saturating light flash (intensity 4). Steady-state ΦII, NPQ, and qL were measured after the plants were exposed to actinic light (80 μmol photons m^−2^ s^−1^) for 10 min. 

### 4.5. Thylakoid Membrane Isolation and Blue Native Polyacrylamide Gel Electrophoresis (BN–PAGE)

Thylakoid membranes were isolated using the method described by Sun et al. (2016) [8]. Arabidopsis leaves were ground in a pre-chilled isolation buffer (400 mM sucrose, 50 mM Hepes-KOH, 10 mM NaCl and 2 mM MgCl_2_, pH 7.8) and filtered through two layers of cheesecloth. The resulting homogenate was centrifuged at 5000× *g* for 10 min. The pellet, which contained the thylakoid membranes, was washed with isolation buffer, re-centrifuged, and finally suspended in isolation buffer. Thylakoid membranes were solubilized in Tris buffer (containing 1% (*w*/*v*) DM in 20% glycerol, 25 mM BisTris-HCl, pH 7.0) for 10 min at 4 °C with 0.5 mg ml^−1^ chlorophyll; insoluble debris were removed by centrifugation at 12,000× *g* for 10 min. The supernatant was mixed with 0.1 volume of 5% Serva blue G in 100 mM BisTris-HCl (pH 7.0), 0.5 M 6-amino-n-caproic acid, and 30% (*w*/*v*) glycerol. A total of 20 μL of this mixture was electrophoresed on a 6–12% acrylamide gradient BN-PAGE gel (according to the manufacture’s info for BN-PAGE gel) to separate the photosynthetic complexes. For immunoblot analysis, proteins were fractionated by 15% SDS-PAGE. Subsequently, proteins were transferred onto polyvinylidene difluoride membranes and probed with appropriate antibodies. Signals were detected by enhanced chemiluminescence (RPN2209, Amersham ECL, GE Healthcare, North Richland Hills, TX, USA).

### 4.6. Positional Cloning by BSA

To generate the mapping population for the *coe2* mutant, plants were crossed to WT Arabidopsis plants of the Landsberg erecta ecotype. A total of 300 *coe2* mutant plants were selected from the segregating F2 population based on high luminescence expression and yellow phenotype. The genomic DNA was extracted and mixed as next-generation sequencing (NGS) sample with the Ler ecotype and Col ecotype’s genomic DNA as control materials. NGS was performed on the Illumina GA IIx platform. Sequencing results were analyzed according to a bioinformatics flow. Based on the genetic linkage rule that the gene mutation and the mutant phenotype are linked, the mutation locus was located on a chromosome. Subsequently, SNPs and INDEL sites in the linkage interval were analyzed, and the difference between Col and Ler was subtracted. Finally, functional mutants that met the genetic rules were identified as candidate genes.

### 4.7. RNA Sequencing and Identification of Differentially Expressed Genes (DEGs)

Total RNA was extracted from the samples using the RNAiso Plus reagent (Takara Biomedical Technology, Dalian, China). Each RNA sample was prepared by adding 1 μg RNA. Sequencing libraries were generated using the NEBNext UltraTM RNA Library Prep Kit from Illumina (NEB, Ipswich, MA, USA), following the manufacturer’s recommendations. Index codes were added to attribute the reads to each sample. Sequencing of the libraries was performed on an Illumina platform and paired-end reads were generated. The raw data (reads) in fastq format were processed with in-house perl scripts by removing low-quality reads which contain adapter and ploy-N. All downstream analyses were performed using clean reads. Gene expression levels were estimated by calculating fragments per kilobase of transcript per million fragments mapped. DEGs between the two comparison groups were identified using the DESeq R package (v1.10.1). The resulting *p* values were adjusted using Benjamini and Hochberg’s approach for controlling the false discovery rate (FDR). Genes with an adjusted *p*-value less than 0.05, as revealed by DESeq, were assigned as DEGs. Three biological replicates were used for RNA-seq. The regulation networks for the TFs and target genes were plotted by Cytoscape according to the PlantTFDB database. The RNA sequence data are available at the (https://dataview.ncbi.nlm.nih.gov/?search=SUB8233125, accessed on 22 November 2021 on https://www.ncbi.nlm.nih.gov, accessed on 22 November 2021).

### 4.8. Gene Ontology (GO) Enrichment Analysis

The enrichment of gene ontology (GO) terms and pathways for the DEGs was analyzed using Metascape (http://metascape.org/, accessed on 22 November 2021).

### 4.9. Grafting Experiments 

The Grafting experiments were performed using the method described by Marsch-Martinez et al. (2013) [63].

### 4.10. RNA Extraction and qRT PCR

Total RNA was extracted with the fastpure plant total RNA extraction kit (Cat. No. DC104, Vazyme; Nanjing, China). Total RNA was treated with DNaseI (Vazyme; Nanjing, China) for 30 min to remove the remaining DNA, then the cDNA was synthesized with HiScript II One-Step RT-PCR Kit (Cat. No. P611, Vazyme; Nanjing, China); qRT-PCR was performed with the corresponding primers (Supplementary Table S5). The qPCR run was performed on a CFX 96 (Bio-Rad) with the following cycle parameter: 95 °C for 30 s, 35 cycles of 95 °C for 30 s, 55–56 °C for 15 s, and 72 °C for 15 s. *Actin* was used as an internal control. Data from three biological and technical replicates were analyzed with Bio-Rad (Hercules, CA, USA) iQ5 software (version 2.0). 

## Data Availability

All data supporting the findings of this study are available within the paper and within its supplementary materials published online.

## Supplementary Materials

The following supporting information can be downloaded at: https://www.mdpi.com/article/10.3390/ijms23020861/s1.
