# Supplementary material for: COE2 Is Required for the Root Foraging Response to Nitrogen Limitation
Source: Int J Mol Sci. 2022 Jan 13;23(2):861. doi: 10.3390/ijms23020861 (PMC8778332; doi:10.3390/ijms23020861)
Supplement: Supplementary file 1 [file ijms-23-00861-s001.zip › ijms-1499701 Manuscript and supplement Table/ijms-1499701-supplementary materials.pdf]

International Journal of
Molecular Sciences

MDPI

Supplementary materials

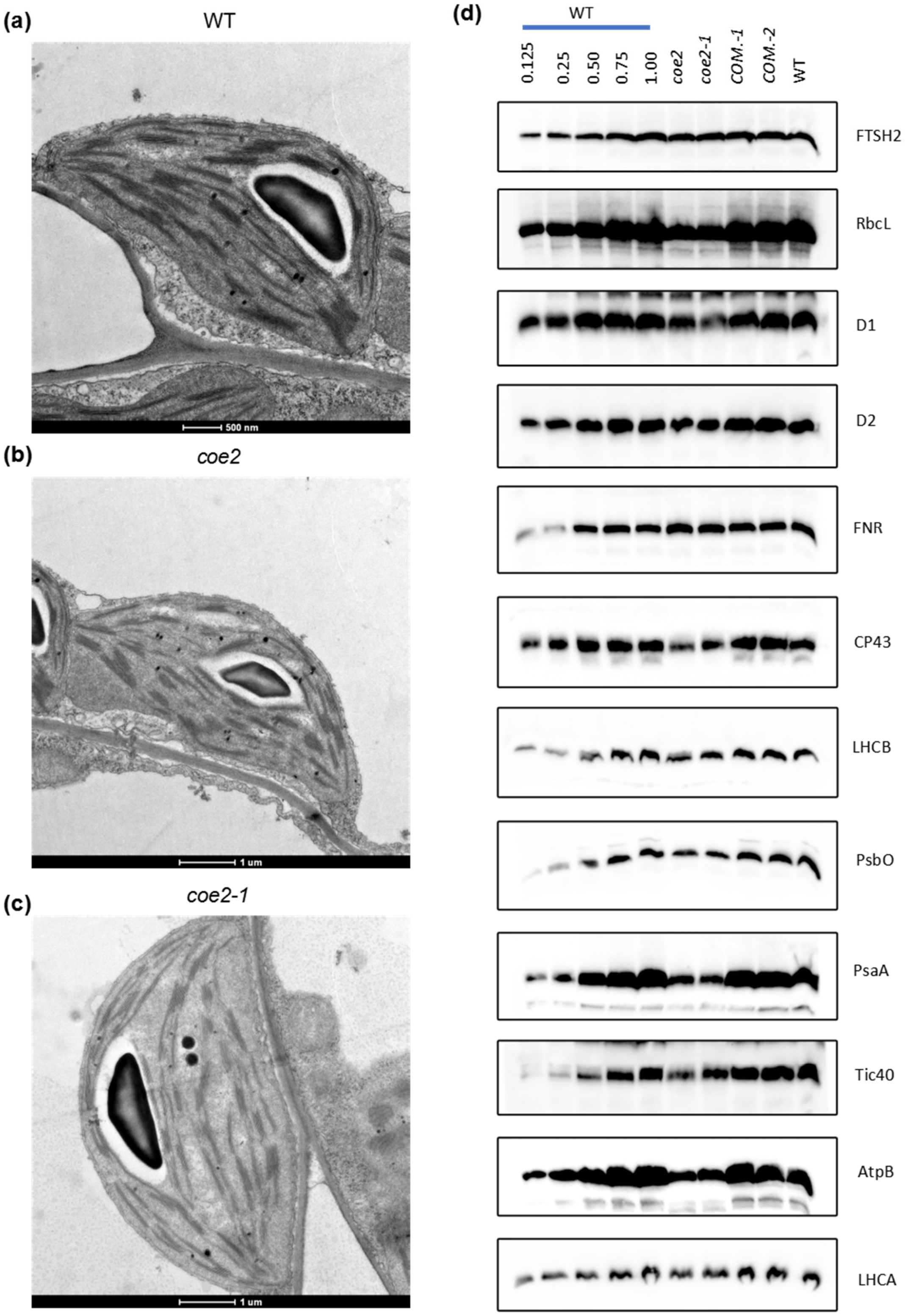

Figure S1. *coe2* is defective in chloroplast development. Chloroplasts of WT (**a**), *coe2* (**b**), and *coe2-1* (**c**) were examined by transmission electron microscopy (TEM). (**d**) Immunoblot analysis of the proteins from photosynthetic complexes in WT, *coe2*, *coe2-1*, *COM.-1*, *COM.-2*. *COM.:* complemented *coe2* line.

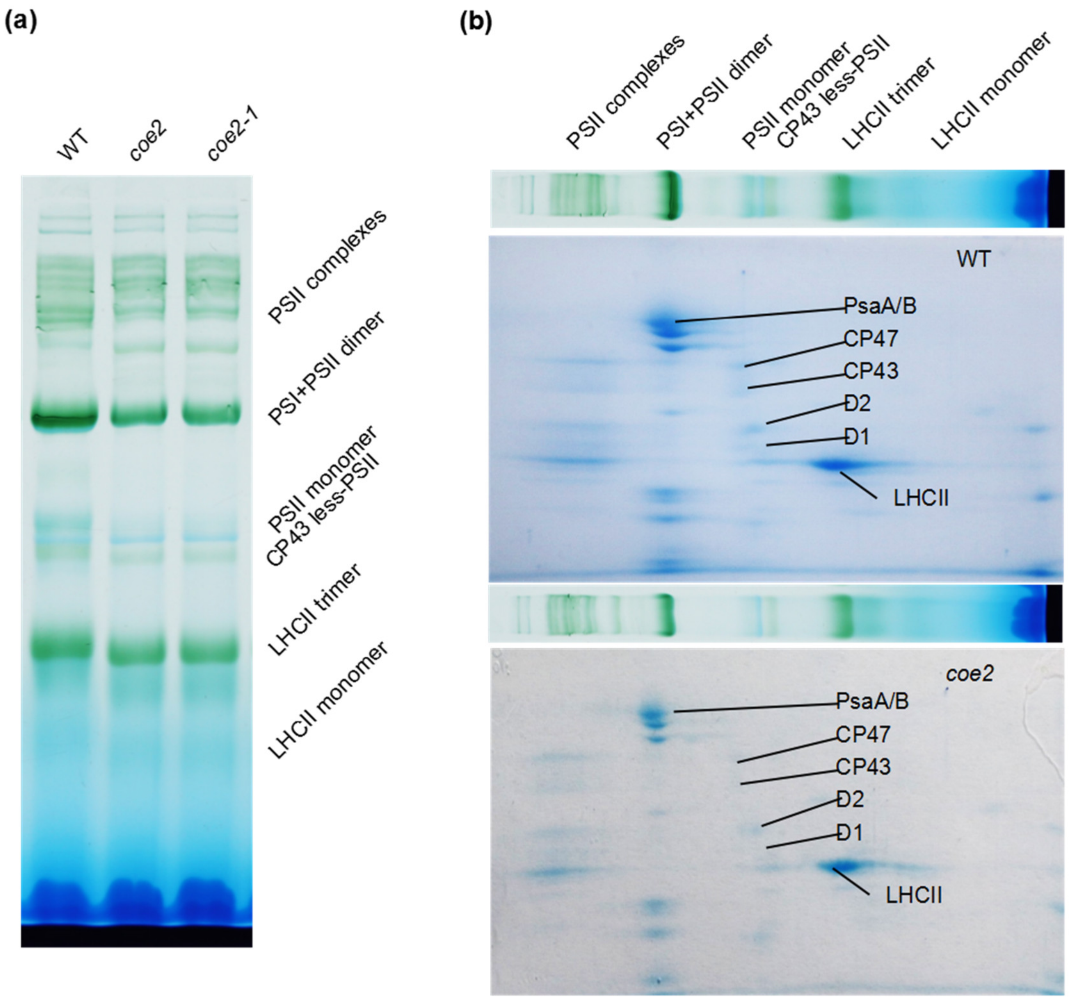

Figure S2. Analysis of photosynthetic complexes in cotyledons of *coe2*, *coe2-1*, and WT by BN gel electrophoresis. (**a**) Thylakoid membranes (10 μg chlorophyll) from wild-type, *coe2*, and *coe2-1* mutant leaves were solubilized and separated by BN gel electrophoresis. The positions of protein complexes were identified with appropriate antibodies. (**b**) BN-PAGE–separated thylakoid proteins were further fractionated by SDS-urea-PAGE. Names of the proteins resolved by the second-dimension SDS-PAGE, previously identified, are indicated.

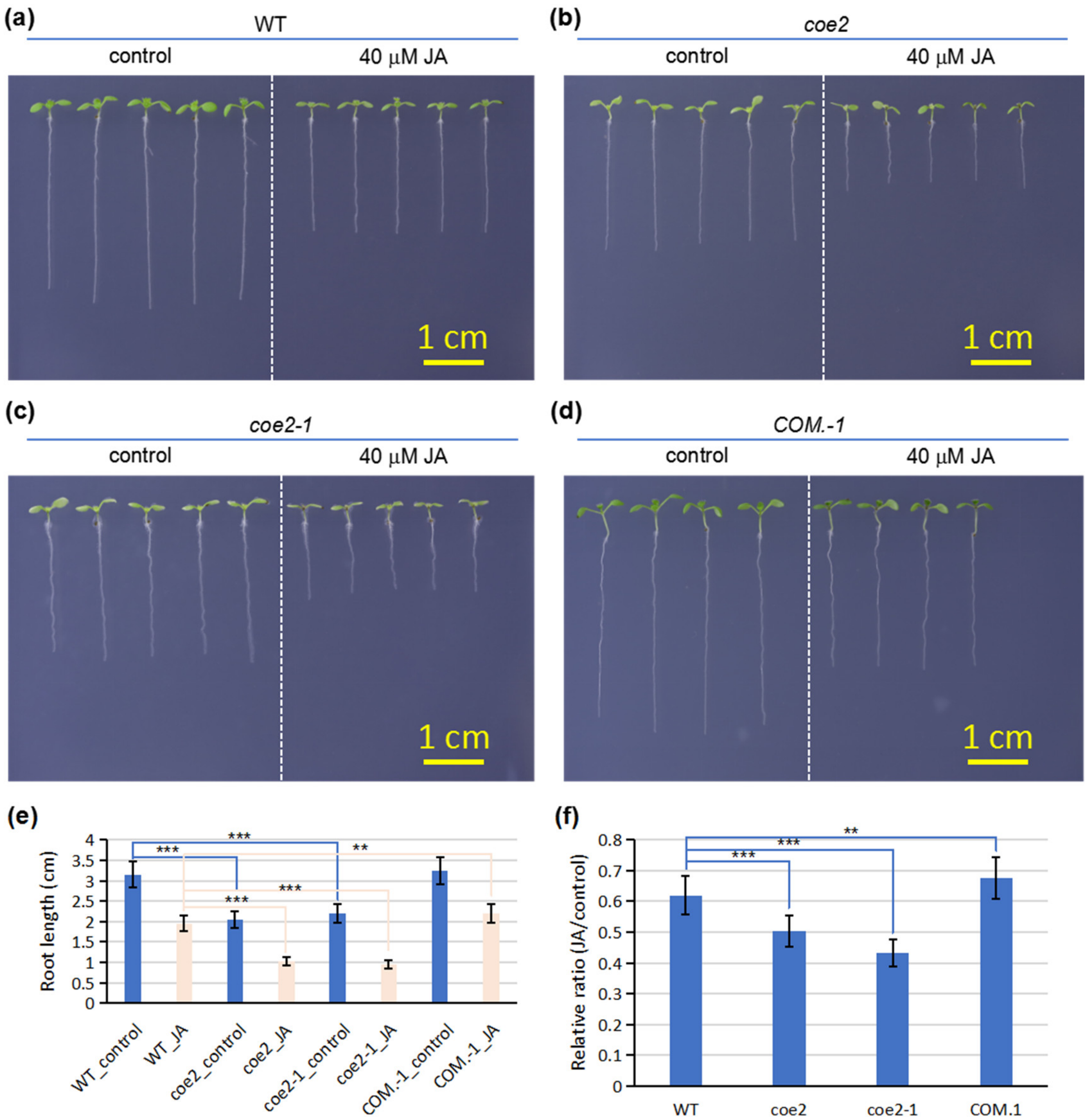

Figure S3. *coe2* mutant shows increased sensitivity to JA. (**a–d**) To monitor the sensitivity of the *coe2* mutant to JA, we examined the effects of JA on the growth of roots of seedlings. (**e**) Statistical analysis of the length of roots of seedlings of WT, *coe2*, *coe2-1*, and *COM.-1* grown under control conditions and with JA. (**f**) Relative ratio of root length of seedlings WT, *coe2*, *coe2-1*, and *COM.-1* grown with JA and under control conditions. Data are given as means (±SD) of three independent experiments. The student's *t*-test was used, $^{**}p < 0.01\%$, $^{***}p < 0.001\%$.

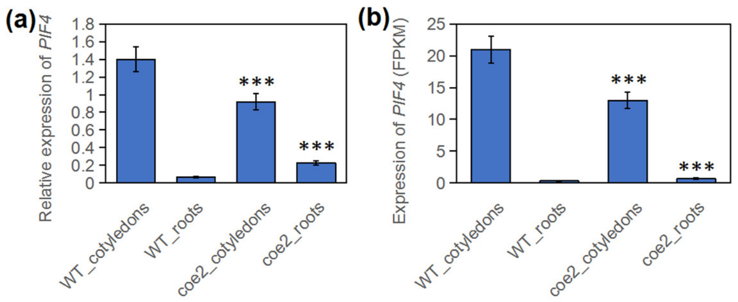

Figure S4. Analysis of the expression patterns of *PIF4* in cotyledons and roots of seedlings. The samples of cotyledons and roots were harvested and used to examine the expression of *PIF4* by (a) qPCR and (b) RNA-seq. 3 biological replicates were used. The student's *t*-test of *coe2* versus WT, $^{***}p < 0.001\%$.

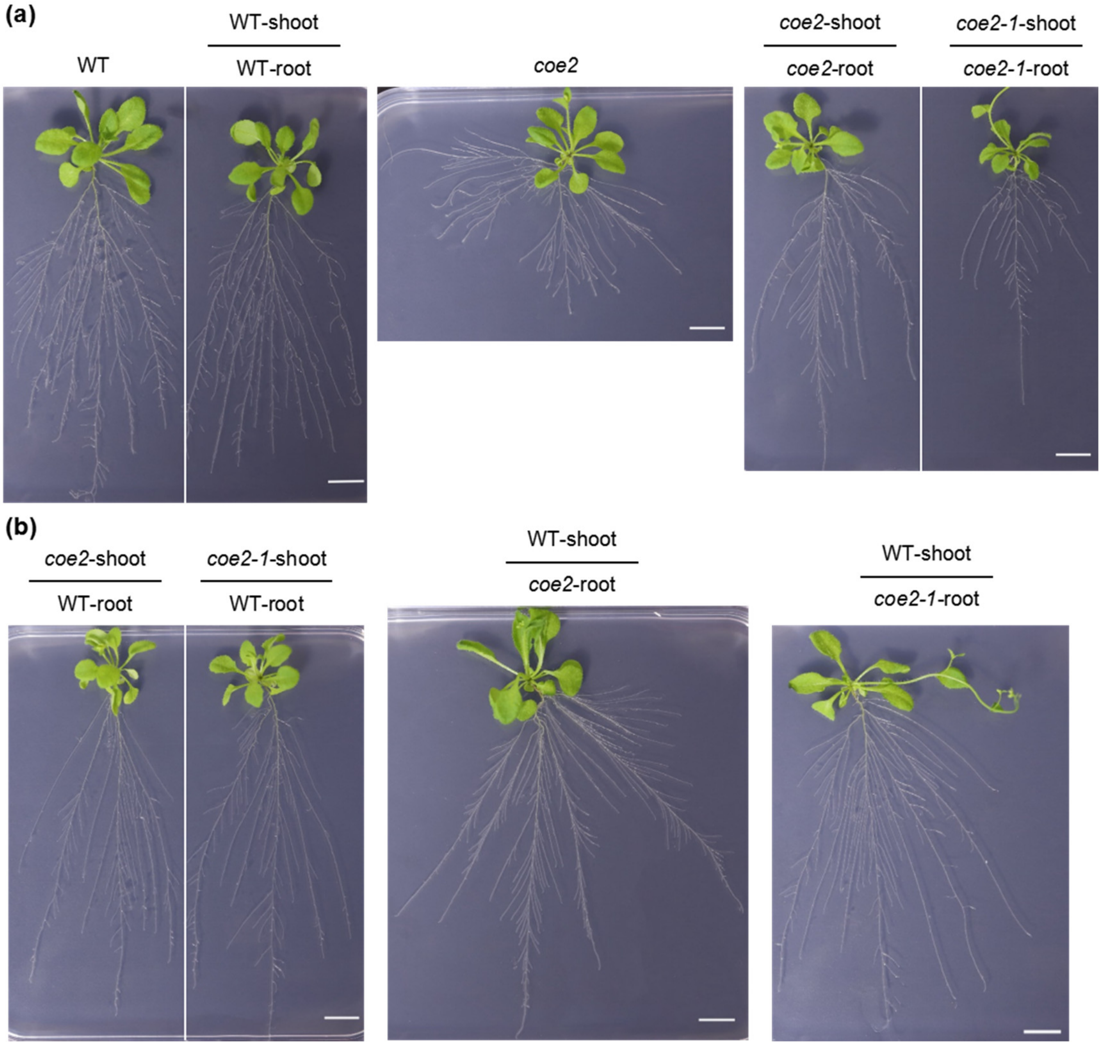

Figure S5. COE2-dependent shoot signaling is required for the regulation of root development. Grafting experiments were performed with the shoots and roots from the same genotype of WT, *coe2,* and *coe2-1*. (b) Grafting experiments were performed with the shoots and roots from the different genotypes of WT, *coe2,* and *coe2-1*. Scale bar:1 cm. The grafting experiments were performed as following: Firstly, prepared two sterile filter papers to a sterile petri dish. Wet the filter papers with 2 ml sterile water. Then, the 3-day-old seedlings that have been grown vertically on ½ MS containing agar plates were used for grafting experiments. Next, under the dissecting microscope, 6-8 very straight seedlings were selected and placed on the top half of the wet filters. After that, use the knife to cut the scion seedlings cleanly, and exactly horizontally, across the hypocotyl just below the cotyledon, and to cut the rootstock seedlings cleanly, and exactly horizontally, across the hypocotyl just below the cotyledon stumps. Then, with the forceps, very gently grab a rootstock piece by the longest root hairs on the oldest part of the root just below the hypocotyl and place it so the cut stump touches the cut stump of a scion cutting. Finally, the reciprocal cuttings can be placed, stump to stump, to form the reciprocal grafts.

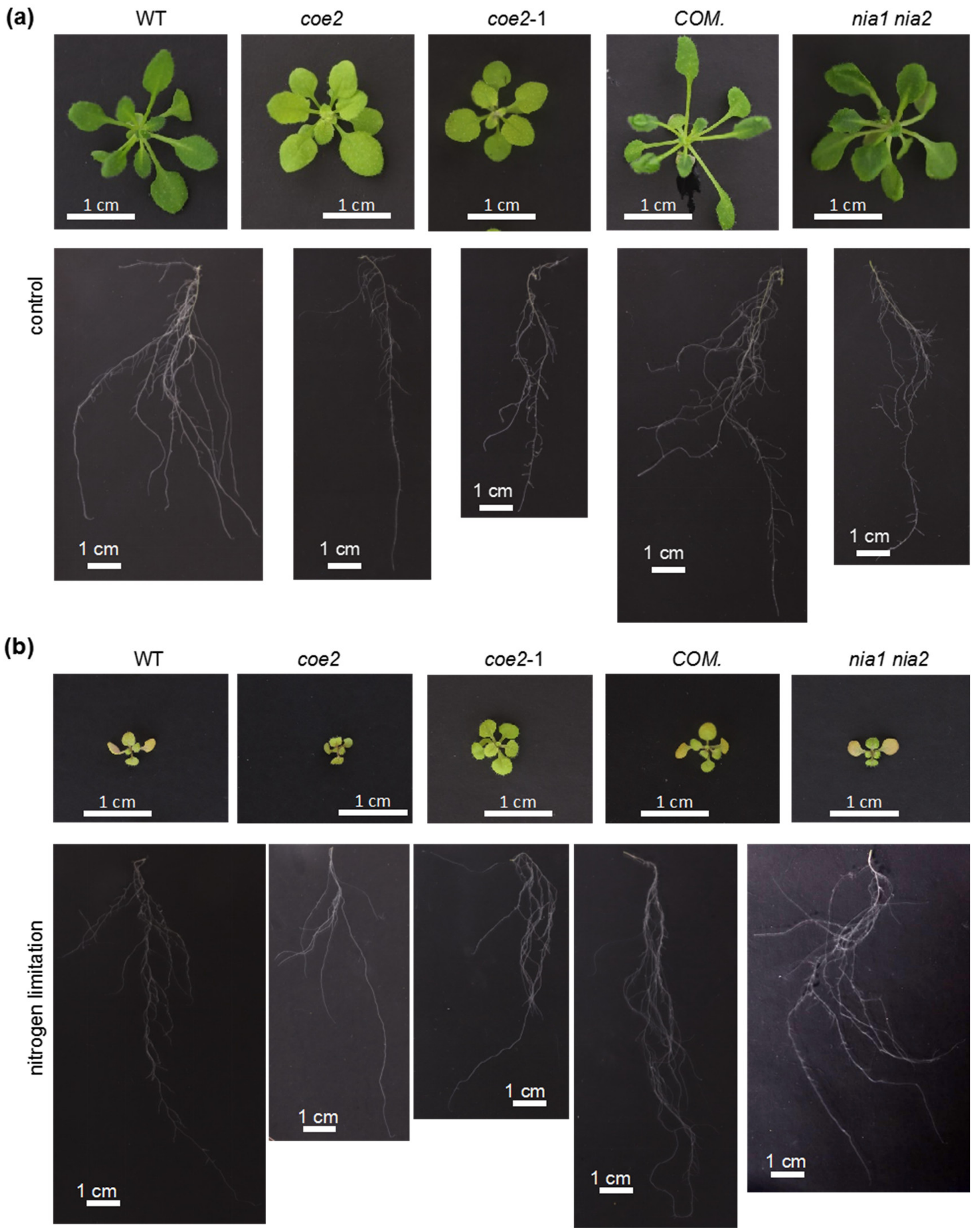

Figure S6. COE2 is required for coordinating the growth of leaves and roots in response to nitrogen limitation stress. (**a**) Growth of leaves and roots of WT, *coe2*, *coe2-1*, *COM.-1*, and *nia1 nia2* grown under control conditions. (**b**) Growth of leaves and roots of WT, *coe2*, *coe2-1*, *COM.-1*, and *nia1 nia2* grown under nitrogen limitation.

Table S3. List of oligonucleotides used in this study.

Gene	Name	Sequence (5' → 3')
		**Plant Transformant**
COE2	COE2s	AAAAAGCAGGCTTCATGGCGCTACGAACACTCTC
	COE2a	AGAAAGCTGGGTGAAAGTACCATTTGGGTCTTAC
	adapter AttB1	GGGGACAAGTTTGTACAAAAAAGCAGGCT
	adapter AttB2	GGGGACCACTTTGTACAAGAAAGCTGGGT
		**Mutant Confirmed**
NOA1	NOA1LP	TTTCCTCATGGTCACTTCCAC
	NOA1RP	TAACCGTCTCCAATCAACCAG
	SalkLB	ATTTTGCCGATTTCGGAAC
		qPCR
NOA1	NOA1rts	CCGGTTACTTCAAATCGAAATC
	NOA1rta	TTTTGGCACACTGACACAAATA
PIF4	PIF4rts	ATTCAGAACAATCCCGGTTTAG
	PIF4rta	GTCTTCGTCGGCACAGACGACG
ACTIN	ACTINs	AACTGGGATGATATGGAGAA
	ACTINa	CCTCCAATCCAGACACTGTA
